# Co-Design of strategies to enhance access to Virtual Urgent Care models by equity-deserving populations

**DOI:** 10.1371/journal.pdig.0001368

**Published:** 2026-04-17

**Authors:** Sander L. Hitzig, Yomna H.E. Ahmed, Akm Alamgir, Courtney F. Kupka, Cliff Ledwos, Rozhannaa Sothilingam, Rhea Alitawi, Marina Motsenok, Johnathon Orr, Logan Reis, Izabelle Siqueira, Sarmitha Sivakumaran, Talitha Brush, Kimberly Devotta, Maarib Kirmani Haseeb, Emily Huynh, Ezza Jalil, Triti Khorasheh, Kosal Ky, Shelley L. McLeod, Wendy MacLellan, Sandra Mills, Katrine Pilested, David Rosenstein, Christine L. Sheppard, Simone Stothers, Jiayu Zhang, Rosanra Yoon, Cynthia Zhang, Shaun Mehta, Umberin Najeeb, Justin N. Hall

**Affiliations:** 1 Department of Occupational Science and Occupational Therapy, Temerty Faculty of Medicine, University of Toronto, Toronto, Ontario, Canada; 2 St. John’s Rehab Research Program, Sunnybrook Research Institute, Sunnybrook Health Sciences Centre, Toronto, Ontario, Canada; 3 Rehabilitation Sciences Institute, Temerty Faculty of Medicine, University of Toronto, Toronto, Ontario, Canada; 4 Dalla Lana School of Public Health, University of Toronto, Toronto, Ontario, Canada; 5 Access Alliance Multicultural Health & Community Services, Toronto, Ontario, Canada; 6 Faculty of Health, Kinesiology & Health Science, York University, Toronto, Ontario, Canada; 7 Department of Equity and Social Accountability, Sunnybrook Health Sciences Centre, Toronto, Ontario, Canada; 8 Women’s College Hospital, Toronto, Ontario, Canada; 9 Family Navigation Project, Sunnybrook Health Sciences Centre, Toronto, Ontario, Canada; 10 Office of the Patient Experience, Sunnybrook Health Sciences Centre, Toronto, Ontario, Canada; 11 Findhelp Information Services, Toronto, Ontario, Canada; 12 Schwartz/Reisman Emergency Medicine Institute, Sinai Health, Toronto, Ontario, Canada; 13 CAMH: The Centre for Addiction and Mental Health, Toronto, Ontario, Canada; 14 Lyndhurst Centre, Toronto Rehabilitation Institute, University Health Network, Toronto, Ontario, Canada; 15 Alzheimer Society of Toronto, Toronto, Ontario, Canada; 16 Michael Garron Hospital, Toronto, Ontario, Canada; 17 Wellesley Institute, Toronto, Ontario, Canada; 18 The Hospital for Sick Children, Toronto, Ontario, Canada; 19 Lawrence Bloomberg Faculty of Nursing, University of Toronto, Toronto, Ontario, Canada; 20 Sunnybrook Health Sciences Centre, Toronto, Ontario, Canada; 21 Department of Emergency Medicine, St. Michael’s Hospital, Unity Health Toronto, Toronto, Ontario, Canada; 22 Division of Emergency Medicine, Temerty Faculty of Medicine, University of Toronto, Toronto, Ontario, Canada; 23 Division of General Internal Medicine, Sunnybrook Health Sciences Centre, Toronto, Ontario, Canada; 24 Department of Medicine, Temerty Faculty of Medicine, University of Toronto, Canada; 25 Department of Emergency Medicine, Sunnybrook Health Sciences Centre, Toronto, Ontario, Canada; 26 Evaluative Clinical Sciences, Sunnybrook Research Institute, Sunnybrook Health Sciences Centre, Toronto, Ontario, Canada; 27 Institute of Health Policy, Management and Evaluation, Dalla Lana School of Public Health, University of Toronto, Toronto, Ontario, Canada; National Tsing-Hua University: National Tsing Hua University, TAIWAN

## Abstract

Virtual urgent care (VUC) was introduced in 2020 across the province of Ontario, Canada, during the COVID-19 pandemic to provide urgent care for lower-acuity health needs for patients who did not require in-person care. Persons from equity-deserving populations (EDPs) often face barriers in using virtual models of care. To raise awareness and improve uptake of VUC among EDPs, a World Café was organized that convened a diverse group of individuals from healthcare, community, research, and/or people with living/or lived experience of health conditions to co-design VUC outreach strategies. The panel consisted of 36 persons (22 invitees; 14 members of the study team) who participated in a one-day, in-person event to reflect on key considerations related to VUC delivery for EDPs through facilitated small and large group discussions. We recorded all discussions via note-takers and analyzed the notes using content analysis. From this analysis, we identified three categories: a) lack of awareness about VUC and accessibility barriers; b) addressing issues of digital literacy and ensuring the perceived credibility of VUC; and c) opportunities for advancing VUC. Given the potential of VUC to serve as a culturally safe modality for EDPs, the World Café event served as a meaningful opportunity to exchange knowledge on health equity and to co-design strategies for improved awareness and uptake of VUC by EDPs.

## Author summary

In 2020, Ontario rolled out virtual urgent care (VUC) to help people with minor health issues during the pandemic, but people from under-served communities, which may include people with disabilities, who are from a visible minority or who are from the 2SLGBTQIA+ community, often struggle to access these services. To address this issue, an in-person meeting was organized where a diverse group of 35 participants from various backgrounds met to brainstorm strategies to improve access to VUC by under-served communities. The meeting attendees focused on key issues such as raising awareness about VUC, improving digital literacy, and making sure people find the service credible. The discussions revealed the barriers people from under-served communities face as well as highlighted opportunities to promote VUC as a culturally safe option for diverse communities. Overall, the event led to several ideas and insights on how to make VUC more inclusive and accessible for everyone.

## Introduction

The COVID-19 pandemic accelerated the adoption of virtual care to ensure access and safety of patients and frontline healthcare professionals during pandemic restrictions and lockdowns, which significantly impacted in-person healthcare access. One such expedited innovation was the advent of Virtual Urgent Care (VUC), developed and led by emergency department (ED) physicians [1]. Sunnybrook Health Sciences Centre, an academic quaternary-care hospital located in Toronto, Ontario, Canada, launched the city’s first adult-focused VUC initiative, which connected patients with low-acuity complaints with ED physicians [1]. VUC models expanded during the pandemic to include other major acute care hospitals in the region, University Health Network (UHN) and Unity Health Toronto. These VUC teams provided care for a wide range of non-emergent conditions, including headaches, body aches, bites and stings, infections (skin, sinus, nail, urinary tract), eye related concerns (eyelid redness or bumps, painless red eye, eye discharge), mild respiratory symptoms (fever, cough, sore throat, nasal congestion), mild gastrointestinal symptoms (nausea, vomiting or diarrhea without severe pain or dehydration), joint sprains, minor wounds and other minor injuries. Patients were able to book same-day appointments through an online platform and connect with clinicians via Zoom (Zoom Video Connections).

Similar to other models of virtual care (i.e., virtual visits with a primary care physician), VUC may be an appropriate alternative for patients with non-life threatening conditions, such as those described above, or as a first step in determining if an in-person visit to the ED is required [2]. In 2024/25, there were 7,301 VUC visits at UHN (n = 2,792) and Sunnybrook (n = 4,509), and similar to provincial reports [2], the majority of cases (92%) were completely managed virtually. As well, the patient satisfaction surveys distributed following these visits found there were extremely high rates of satisfaction (95%) with the service, with most surveyed patients indicating it was better than in-person care (92%), that the technology was easy to use (96%), and that they would recommend VUC to others (95%).

Despite its many advantages, not all communities have access to virtually delivered healthcare programs [3–5]. A health services data analysis of VUC use at Sunnybrook found that patients accessing the service tended to have completed higher levels of education (university degree or higher), be middle aged, were female, English-speaking, and urban-dwellers of high socio-economic status [2]. Consequently, there are on-going concerns regarding the ability of persons from equity-deserving populations (EDPs) to access VUC [2,6]. According to the Digital Health Equity Framework, access to virtual supports is influenced by an interaction of socio-economic and cultural factors, including social support, behaviours and beliefs, and the environment [4]. Common barriers to virtual care modalities include lack of Internet access, lack of a private space to receive care, low digital literacy and non-English language preferences [4,5,7].

Between 2022 and 2023, a series of qualitative studies were undertaken to better understand the VUC experiences of patients from EDPs [6], and ED physicians’ perspectives on providing virtual care to patients from EDPs in the Greater Toronto Area (GTA) [8]. In the study that explored patient experiences with VUC, 36 patients and 7 family caregivers from diverse backgrounds shared how VUC was viewed as much more culturally safe than their in-person ED visits. Participants described how VUC provided more time for physician-patient interactions, and allowed the patient to have more control over the care experience (i.e., having a family member attend) [6]. Similarly, ED physicians (n = 14) working at three different VUCs across the GTA shared how this virtual modality offered patients an accessible and inclusive space for receiving care compared to their in-person ED experiences, which enhanced patients’ autonomy [8]. Both patients and physicians from these studies described the need for increased awareness about VUC to different communities to promote uptake, and to develop tailored strategies to optimize delivery [6,8]. Engaging with community health and social care support providers in different patient populations (e.g., unhoused, immigrants and refugees, persons with disabilities, etc.) was identified as one way to raise awareness [8].

Over the past three years, VUC in Ontario has undergone changes, with some sites closing (i.e., Unity Health, Toronto), while other programs have evolved in terms of their structure. For instance, VUC at Sunnybrook (known as the Virtual ED) expanded its service providers to include nurse practitioners as the first point of contact in care interactions and extended the hours of service. Similarly, the program has integrated full translation services to make it more accessible to those with non-English language preferences. Other efforts include developing collaborations with regional partners, integrating VUC with Health811 (remote access to a registered nurse in Ontario), and further development of hybrid models of care combining in-person and virtual care.

As the landscape of VUC in Ontario continues to evolve, the experience and barriers for under-served communities must be considered [8,9]. Hence, the purpose of this study was to engage a diverse set of key interest groups to co-design strategies to enhance access to the VUC for EDPs in the GTA.

## Materials and methods

A modified World Café approach [10] was selected to facilitate the co-design of strategies to enhance access to VUC for EDPs. A World Café is a creative process for engaging participants in table discussions around certain topics for a short amount of time [10]. This qualitative research approach [11] can be regarded as a group discussion, whereby sub-research questions are assigned to different tables, which has participants randomly rotate to build on the generated knowledge to discuss each sub-question in small groups. In our approach, we did not rotate participants (described in the procedure section below).

### Ethics statement

Ethics approval for this World Café event was provided by the Research Ethics Board at the Sunnybrook Health Sciences Centre (ID#5080). Written consent was obtained from all participants.

### Participants

For this event, our team adopted a broad definition of EDPs, which were any persons who faced systemic barriers to potentially accessing and using virtual models of care. Populations targeted included, but were not limited to, the disability community, the 2SLGBTQIA+ community, racialized communities, immigrants or refugees, and persons who were unhoused.

Participants in the World Café were adults (18 years and older) who fell into three – at times overlapping – groups: a) members of an EDP who had previously received virtual care of any type; b) family/caregivers of members of an EDP who had received virtual care of any type; and c) health or social support professionals (i.e., ED physician or nurse practitioner, hospital or community provider/decision-maker, community advocate, researcher, etc.) with relevant knowledge regarding EDPs and/or virtual models of care.

We identified potential World Café participants through the collective networks of our investigation team, which included connections across hospitals providing VUC, community health centres, research institutes, and municipal and provincial organizations. Through these networks, organizational partners and care providers assisted with outreach to identify a diverse set of persons with lived experience who had received and/or provided virtual care to participate in the World Café. From these various sites, and through snowball recruitment methods [12], we identified a diverse group of 70 participants.

### Procedure

Our team sent identified participants an invitation letter and a link to an online survey that contained the informed consent form. After participants provided their written consent to participate in the World Café, they were asked to complete a brief socio-demographic survey to self-report data about their EDP status, and professional role if applicable. Based on identified status (i.e., professional – community; professional – hospital; person with lived experience), attendees were assigned to small groups with the goal to maximize diverse perspectives on the topics to be discussed throughout the day. The World Café occurred at an accessible community centre in downtown Toronto on August 13^th^, 2025, and was structured to facilitate the exchange of knowledge regarding the VUC needs of EDPs.

For the first part of the World Café, attendees were provided guiding principles to optimize participation while also ensuring personal safety and well-being (see Table 1 for list of principles) [13].

**Table 1:** World Café Guiding Principles.

Guiding Principle	Description
Approach Listening with Openness and Respect	Listening to others is an important way to acknowledge differing perspectives and viewpoints. Aactive listening means thoughtfully considering the ideas being raised by others. Try to listen for understanding, and to suspend judgment when listening, and be mindful of any judgments that do arise.
Acknowledge and Respect Difference	Each person’s experiences are shaped by their identity, positions, and their relationship to interconnecting systems and structures of power and inequity.
Disagree with the Idea, Not the Person	Be open to receiving multiple perspectives and have respect for differences of opinions.
‘Take Space/Make Space’	Reflect on how you generally participate in a group discussion. Consider what you can do to balance your own participation in the conversation while also creating space for others to contribute.
Speak from Your Own Position and Experiences	Talking for someone else can invite unhelpful assumptions into discussions. Try to speak from the ‘I.’
Witnessing is Participation	No one is required to do anything that does not feel right to them, which includes sharing their stories or suggestions. Witnessing is a form of participation and is viewed as a means of contributing to the conversation.
Maintain Confidentiality	Attendees should not share anyone else’s personal stories unless they have express permission from the other person. Since attendees may also be part of a team, or work together, it is especially important that participants not feel that what they share in a good faith process of learning (e.g., personal struggles) be used against them outside of this space.
Take Care of Yourself	Remember to do what you need to do to take care of yourself (e.g., take a break, drink water, seek out support, etc.).

The next part of the event included an overview of VUC provided by the Director of the VUC program at Sunnybrook (JNH, senior responsible author), which detailed how it was developed, the scope of practice (i.e., types of ideal conditions that could be treated virtually, populations served), visit outcomes, patient experience, and long-term goals for expansion. As well, two researchers (SLH, YHA) provided a summary of qualitative findings from a study that explored the perspectives of EDPs who used VUC services [6], and another study that explored ED physician perspectives on providing VUC to EDPs [8]. The key highlights were both patients from EDPs and ED physicians viewed VUC as offering culturally safe care in comparison to in-person ED care. While both groups acknowledged limitations of virtual care (e.g., unable to undertake a complete physical exam, technology issues, etc.), they also identified several advantages over in-person emergency care, including more time with the treating physician, scheduled appointments, and no travel. Finally, participants across both studies highlighted the need to promote greater awareness of the service and provided suggestions for improving access and uptake by EDPs [6,8].

Following these presentations, three separate small group discussions were organized on the following topics:

1) Reactions and reflections regarding VUC and the presented research findings on VUC delivery for EDPs;
2) Generating suggestions for delivery of compassionate and culturally safe VUC for EDPs; and
3) Developing appropriate outreach strategies for raising awareness about VUC to improve uptake by EDPs.

**Fig 1:**
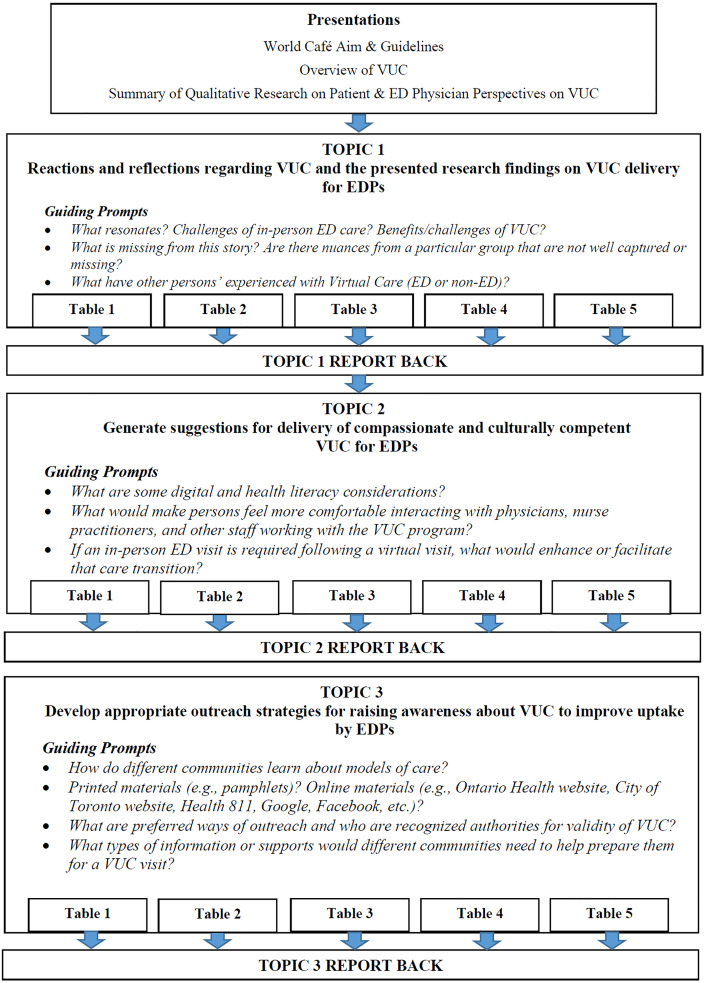
World Café Structure.

Each table had a facilitator to guide the small groups, and projector screens in the room provided relevant prompts to focus the discussions (see **Fig 1**). Facilitators used large notepads at each table to document key ideas discussed. Additionally, each table had a note-taker who documented the small group discussions using a Word document on a laptop as well as key ideas exchanged during the large group share-back sessions. Given the professional and/or lived experiences of the facilitators and note-takers, they were also encouraged to provide insights into the topics discussed. The points written on the large notepads were cross-referenced with the note-takers’ documents and integrated for analysis following the meeting.

For our World Café small group discussions, a modified approach was adopted to account for several logistical factors (i.e., time, space, mobility impairments of attendees, etc.) while maintaining mechanisms for knowledge exchange that could be collectively built upon. Specifically, World Café attendees remained at their assigned small tables throughout the day as they discussed each subsequent topic related to VUC rather than having attendees rotate to different tables. However, we built in opportunities for each group to share back to the larger set of attendees by having the facilitators at each table refer to the notes on the large notepads to recount issues discussed in the small groups. The full set of attendees were also offered time to provide additional commentary or ask questions during these share-back sessions. Another mechanism to facilitate transfer of collective knowledge was to have each table facilitator rotate across World Café topics, which enabled them to share key learnings with other groups over the course of the event. Finally, two moderators (SLH, AA) circulated throughout the small groups over the course of the day to ensure discussions stayed on track, and to offer some insights into the small group discussions where appropriate (i.e., clarifying information about the VUC, research findings, etc.).

At the end of the day, our team thanked participants for their time and participation. A $50 stipend was provided to persons with lived experience, and all participants were provided lunch and refreshments throughout the event.

### Analysis

The data for analysis were drawn from the notes recorded on the large notepads, the documentation by the note-takers during the small group discussions, and note-takers’ documentation of ideas raised during large group share-back sessions. While bias cannot not be eliminated given the structure of the World Café, the use of the notepads to document ideas, and triangulating these notes with the note-takers’ documentation served to minimize bias. A qualitative content analysis [14] was undertaken to organize the discussion notes [15].

To achieve this goal, three reviewers (YHA, SLH, RS) independently reviewed the transcribed notes to summarize key ideas discussed at the World Café. Patterns of discussions were independently coded by two coders (YHA, RS) and were reviewed by a third coder (SLH) to organize and label the categories. This process took place in two rounds of review until a coding framework could be agreed upon. The categories were identified and summarized once a high level of agreement was achieved, where both raters were coding the same text similarly. The findings were shared back to the World Café attendees for verification, which served as a member check; thereby enhancing the trustworthiness of the data [16].

## Results

Of the 70 identified potential attendees invited to the World Café, 24 agreed to attend and provided consent, and 22 attended in person. The 14 members of the investigation team who participated and supported the World Café had expertise in ED care, virtual models of care, and/or the health and social care needs of EDPs. Hence, a total of 36 persons participated in the World Café event. Table 2 provides an overview of the participants, which includes persons with overlapping (i.e., professional and/or person with lived experience) and intersecting identities.

**Table 2:** World Café Attendee Characteristics (N = 22).

Role	Frequency
Healthcare provider*	7
Advocacy or non-governmental representative*	4
Researcher	5
Other professional*	3
Person with lived experience	3
**Age Group**	
18 to 29 years	3
30 to 64 years	18
65 years and older	1
**Racial or Ethnic Group**	
Asian - East (e.g., Chinese, Japanese, Korean)	4
Asian - South (e.g., Indian, Pakistani, Sri Lankan)	5
Black - African (e.g., Ghanaian, Kenyan, Somali)	1
Middle Eastern (e.g., Egyptian, Iranian, Lebanese)	1
White - European (e.g., English, Italian, Portuguese, Russian)	10
Other	1
**Gender**	
Man	4
Woman	18
**Sexual Orientation**	
Heterosexual	20
Asexual	2

*Please note that some attendees indicated they also had lived experience of being from an EDP or undergoing virtual care; Only reported options are presented in the table.

From the discussions at this event, findings were organized into three categories: a) lack of awareness about VUC and accessibility barriers; b) addressing issues of digital literacy and ensuring the perceived credibility of VUC; and c) opportunities for advancing VUC. While many of the issues described have interconnected or overlapping features, the categories summarizing the findings are organized to highlight key concerns and link them with actionable strategies to enact positive changes at the patient-, practice- and system-levels.

### Lack of awareness of VUC and accessibility barriers

Several attendees discussed how there is a lack of awareness about the existence of VUC, which included key interest groups who are generally well-connected and involved with the healthcare system. This prompted discussions on the importance of raising awareness of the different roles of the VUC team and emphasized a cultural angle to how patients understand certain roles. For instance, some attendees noted that members of certain cultural groups may be unfamiliar with the need for an official referral to see specialists. As well, they may not understand the nurse practitioner role, as this role does not exist in many countries, which can lead to confusion when they do not directly interact with a physician while using the service.

Relatedly, attendees underscored the importance of raising awareness about the scope and goals of VUC, which is to clarify the types of complaints or issues that are appropriate for this care modality. Specifically, attendees stressed that when raising awareness about the service, it should be framed as an opportunity to clearly explain when and how patients should use VUC rather than solely as a means for diverting patients from an in-person ED visit. Similar to EDs, which are facing tremendous strain, there were concerns that VUC could also become overburdened if demand grows without adequate support. Attendees pointed out the important role of other healthcare providers in raising awareness and proper uptake, which could be through family physicians, having ED physicians educate their patients about the service during their in-person ED visits, and by building partnerships with other primary care providers. Participants also discussed how common community channels such as schools, libraries, and shelters could potentially serve to facilitate access for VUC for harder to reach populations. Regardless of the sources of outreach, materials should be tailored for the EDP community in mind, and obtaining feedback from these communities was important to refine outreach strategies to meet local needs.

Additionally, there were discussions about the need to raise awareness about VUC for persons with limited mobility, or who have unreliable means of transportation. For instance, wheelchair users might be a good group that VUC should be promoted to since it was noted that this population uses the ED as their primary means of care due to the lack of accessibility of family physicians’ offices or their physicians’ general lack of knowledge on how to best serve the disability community. As well, attendees highlighted that people experiencing intimate partner violence or living in unsafe home environments were a particularly vulnerable population since they may lack access to safe and reliable transportation to reach an in-person ED or who may face surveillance and control by partners that further limits their ability to seek care. There were also discussions on how to make VUC more accessible to residents of long-term care homes, who often have complex medical needs, mobility limitations and high reliance on staff for transportation.

Another important barrier to awareness and uptake of VUC is language and interpretation. Attendees stressed the need for clearer, multilingual, and culturally sensitive communication approaches in VUC. There was general agreement among attendees that the consistency and quality of interpretation services vary across different care settings (i.e., not specific to the VUC setting), which can lead to miscommunications between patients and providers; thereby leading to poorer care experiences (i.e., feelings of frustration, suboptimal care). One attendee recounted how a newcomer from the Bengali community had a very stressful in-person care experience due to the poor quality of the hospital’s interpretation services. Some additional considerations raised were that patients may feel more comfortable with a family member than a hospital interpreter and might even avoid appointments if they do not feel comfortable with the interpreter provided. Offering advance information about whether an interpreter will be present might be especially helpful for communities who have historical mistrust in the healthcare system. Other noted accessibility barriers to using VUC were related to patients with sensory impairments, such as those who are hard of hearing or who have low vision, who may physically struggle to navigate the online landscape.

### Digital Literacy (Digital Divide) and Credibility

Attendees discussed how the focus of VUC remains hyperlocal and is utilized by a small set of users from specific postal codes. Meaning that despite the promise of the technology not being limited by geography, the service was primarily being accessed by urban-dwelling individuals from higher socio-economic statuses and with higher levels of digital literacy (e.g., no or minimal challenges with logging into platforms, use of passwords or filling online forms). Hence, there were concerns that VUC cannot meet the needs of those outside of the hospital’s catchment area, such as rural-dwelling individuals, or those EDPs known to have barriers to digital care modalities, including older adults, persons with disabilities (e.g., physical, sensory, cognitive, etc.), newcomers, and unhoused individuals and populations experiencing lower digital literacy. In general, there was a sense that there is likely a lack of representation of persons from those communities using VUC due to a lack of access to the Internet, devices, or confidence in using virtual platforms.

Additional considerations raised with virtual platforms included the varying degrees of comfort that people had using a phone, Zoom, or other platforms, with attendees stating that users preferred using technologies they were familiar with. Addressing this variability, from both a provider and patient perspective, requires further reflection and planning to minimize confusion, which might serve to facilitate greater willingness to access and use VUC.

In terms of patient privacy, discussions revealed that patients may be concerned about being overheard at home while disclosing sensitive information, and feel uncertain about confidentiality online, which can negatively affect their comfort in sharing their reason for seeking medical attention, especially for stigmatizing health concerns. One situation mentioned where this may arise was for persons living in multigenerational households (e.g., South Asian community), where family members may participate in the patient’s care encounter or may overhear the visit because there is no private space in the home. To address these concerns, attendees suggested offering details ahead of the appointment about privacy considerations in a VUC encounter (e.g., range of information that is typically shared in this setting), who can be present (e.g., family caregiver), and what type of setting might be ideal for a VUC appointment (e.g., try to identify a safe and secure place if possible).

Relatedly, it was recommended the online intake forms have open-ended fields and have transparent explanations on why certain information is required to encourage disclosure, which could serve to reduce patient anxiety on how their data would be used during VUC visits. It was discussed that these fields could also be used for patients to share more privately relevant information but signal that it is not safe to discuss it during the appointment. Hence, a clear and appropriate explanation of the purpose of the types of questions being asked and collected was viewed as being critical to help build trust with certain communities.

Digital literacy challenges were also highlighted in three main situations: when there were unclear or long disclaimers when accessing the virtual appointment; when there was missing contact information; or when there were untrusted links (i.e., legitimate but unfamiliar descriptions to patients). Attendees felt these issues eroded trust in the VUC system by patients, particularly when they may not know who to contact with questions about how their ‘digital footprint’ would be recorded, how their data would be used, or by whom. Hence, patients with low levels of digital literacy may become overwhelmed with the online information on a VUC portal (or on other sites detailing VUC services). To promote confidence in virtual models of care, there is a need for strategies that can accommodate varying levels of digital literacy

Building on earlier discussions regarding pre-appointment guidance detailed above, one mechanism to build confidence in using VUC would be the use of trusted community hubs that could potentially host VUC kiosks with administrative support to help patients book and prepare for an appointment, serving as a point for VUC access in the community. These alternative points of access, which could include the use of phones and printed forms, could help ensure equitable access to VUC by providing patients with safe and trusted environments for use.

Overall, trust and credibility were repeated challenges that needed to be addressed to enhance VUC use by EDPs. Some attendees reported patients often questioned whether virtual care is legitimate, reflecting the general patient perception and skepticism toward online healthcare. Meeting attendees described a general distrust or stigma surrounding virtual care—associating “virtual” with lower quality or higher risk. This was tied to growing concerns over artificial intelligence in online scams, and that in-person care was seen as the superior care modality. Hence, having clear, consistent branding from widely recognized hospital or government bodies linked to the VUC would help establish its credibility, and is critical for indicating that the VUC is a legitimate, trustworthy care option.

### Opportunities to Advance VUC

Attendees emphasized the importance of identifying and engaging EDPs who are currently underrepresented in VUC. As previously described, newcomers, uninsured patients, unhoused individuals, and older adults are likely experiencing hurdles when trying to access VUC. One recommendation was to undertake further research to better understand which groups were not accessing VUC, and to identify ways to improve access given that it could serve as a key access point to the health system, with discussions to further investigate this for newcomers and immigrants. Another group identified as having significant barriers to access for the VUC was people who are unhoused or struggle with substance use. Overall, there remain critical gaps in knowledge about certain EDP communities and their specific barriers to accessing the VUC, highlighting an opportunity to further explore VUC for patients from diverse backgrounds

Attendees also discussed how providing guidance and training to VUC providers could enhance the patient experience. Some concrete examples provided included the use of plain-language communication, structured teaching and scripts, inclusive and culturally competent practices, and ongoing evaluation to ensure compassionate, accessible, and patient-centred VUC delivery. While preliminary findings on EDP experiences with VUC indicate that this type of care experience is characteristic of their virtual care visits compared to their in-person ED experiences [6], World Café attendees emphasized there are opportunities to further explore ways to integrate cultural safety and trust-building practices within ED care across both in-person and virtual settings to better meet the needs of EDPs.

Another opportunity discussed was to develop a partnership database to standardize VUC as a unified resource to streamline referrals, ensure consistent care and improve equitable access. In some cases, this could be partnerships with healthcare systems (i.e., Ontario Health Teams; consortiums of hospital and community providers working collaboratively to provide care to specific regions of the province), or with standalone community providers. Moreover, standardized VUC platforms with integrated communication systems could reduce confusion, improve coordination, and ensure seamless, and continuous care for patients from under-served communities; many of whom often face fragmented interactions with the healthcare system.

Given that VUC is a digital care modality, there was some discussion among attendees about the potential use of artificial intelligence to enhance accessibility and knowledge dissemination by simplifying medical information into plain, multilingual language, improving digital usability through user-friendly methods and enhancing reach via QR codes, search integration, and automated translation tools. Additionally, participants discussed creating an online self-triage process to better direct patients on where they should seek medical care. These online tools could include automated symptom checkers (i.e., fever, cough, rash, etc.), and educational resources to allow patients to independently determine whether to seek care through VUC or attend the in-person ED.

In terms of specific populations, there were discussions about the needs of persons with mental health issues and/or substance use disorder, with additional considerations for the unhoused members of this population. It was suggested to explore ways in which VUC could connect with non-medical detox and social service settings with virtual medical oversight. Attendees felt this connection would support continuity of care for this population and help to reduce unnecessary in-person ED visits. In some cases, there might be a possibility of using VUC to improve stability in patients with mental illness or substance use disorder and de-escalate crises that might otherwise intensify in a busy and overstimulating in-person ED setting.

Attendees also reflected on the possibility of extending VUC coverage hours. This included offering walk-in options (as opposed to pre-booking an appointment), which would be essential for patients with after-hours or irregular care needs, such as those patients who find scheduling difficult (e.g., unhoused community). Also, the attendees agreed that VUC might not always be appropriate for every medical issue but could serve to complement in-person care. This was related to using VUC to promote continuity of care where records of health interactions could be synchronized to support efficient and ‘warm’ handovers from one care modality to another; thereby preventing patients from having to repeat triage or come back in-person and experience long wait times. Similarly, there were discussions emphasizing the potential of VUC to improve referral and follow-up pathways, helping ensure that patients are promptly and appropriately linked to specialized or ongoing care after their virtual visit. The potential of expanding VUC to the wider interprofessional team was also discussed, whereby other health professions could provide VUC depending on the reason for the visit and their needs presented, which may require specialist opinion. For instance, attendees highlighted that patients sometimes come to the in-person ED to access a social worker.

Overall, attendees emphasized the need for clear, compassionate, and accessible communication in VUC, including clear pre- and post-visit guidance, plain-language follow-up notes, and structured, scheduled appointments to enhance comfort, predictability, and patient-centredness. Doing so could promote higher levels of self-management in patients and enhance their sense of autonomy and their confidence in directing their care.

## Discussion

The findings from the present World Café expanded upon previous work on VUC for EDPs [6,8] by providing deeper insights regarding: a) lack of awareness about VUC and accessibility barriers; b) issues of digital literacy and the perceived credibility of VUC; and c) opportunities for improvement. Overall, the issues discussed across these three domains provide more nuance on the VUC experience for EDPs.

Regarding the credibility of VUC, many of the participants highlighted the need to ensure it is clearly recognized as a legitimate and trustworthy source, noting that many patients and clients are hesitant to engage with online platform due to fears of online scams. The College of Family Physicians of Canada recognizes that with the emergence of virtual models of care, there are also for-profit organizations offering virtual care, where companies may promote ordering unnecessary tests and advertising directly to both patients and providers [17]. There are also several nefarious online schemes as it relates to telehealth, such as in the United States, where the National Health Care Fraud Enforcement Action pursued felony charges against 193 defendants across the country in 2024 [18]. Additionally, the growing use of artificial intelligence in online fraud [19] is contributing to feelings of mistrust with virtual care. As such, many attendees stressed the need for clear branding that explicitly links VUC to its home organization, the Sunnybrook Health Sciences Centre, which is well-recognized and leading hospital in the GTA, to help reassure patients of its legitimacy. Although these issues are not specific to EDPs, it may be of greater importance to inform persons from vulnerable sectors what to look for to ensure they have engaged with a reputable virtual care modality.

Relatedly, World Café attendees discussed how some of their patients/clients were concerned about their privacy. In particular, attendees discussed how some of their patients/clients lived in multi-generational homes and highlighted that if family members were to be present during a VUC visit, it would be critical to ensure that questions about their health concern be asked in a culturally appropriate and non-stigmatizing manner. In other instances, there may be mistrust on how data collected during the healthcare interaction may be stored or used. One study noted that mistrust is an identified barrier to accessing digital health interventions among undocumented individuals due to concerns that the collected data from the online platforms that be used to put them at risk for deportation [20]. At our World Café event, additional concerns about using VUC were related to patients who do not have public-system health insurance (i.e., undocumented, refugees, etc.). A qualitative study on the in-person ED use of non-insured patients reported that participants felt unwanted and powerless, and faced challenges in navigating the healthcare system and accessing care [21]. Participants felt stigma and discrimination, which are concerns likely experienced by other EDPs [21]; all of which can erode trust in the healthcare system. As such, by listening to patients from under-served communities and integrating their feedback into virtual care design, many issues that often arise in in-person encounters may be mitigated more effectively in a virtual setting [6]. However, there remain a number of factors that should be taken into consideration for building trust in terms of what data is captured by VUC, and how it is used.

To enhance the patient experience and to foster greater levels of trust with VUC, there is a need to ensure that patients have appropriate support prior to booking an appointment so that they can ensure they understand the implications of using VUC (e.g., where to book, simplifying language rather than using medical jargon, etc.), and how to best prepare for an appointment. While there is an opportunity to develop resources to support patients from EDPs to use VUC, there is unfortunately limited evidence on interventions for improving e-health literacy in under-served populations [22]. Consequently, it is imperative that any new resources be co-designed with the relevant community to maximize their effectiveness and acceptability to improve access to VUC.

Importantly, VUC providers should also undergo the appropriate training to best meet the diverse needs of their patients. Several reviews undertaken by Hilty and colleagues [23–25] have outlined cultural competencies for providers that need to be taken into account with regards to telehealth interventions, and categorize these considerations by provider levels of expertise (novices, experienced, and experts). While there is initial evidence on the importance VUC physicians place on ensuring patients from EDPs receive compassionate care [8], the resources and categorizations outlined by Hilty and colleagues may offer an additional resource to build cultural competency in VUC providers in a more structured and systematic fashion.

In terms of raising awareness and access, issues that were explored in our previous research [6,8] were re-visited. Proposed solutions included using a variety of outreach strategies tailored to the targeted community (e.g., pamphlets, translated materials, use of social media to promote VUC). Importantly, it was shared that many of the attendees were not fully aware of the existence of this service and highlighted how the World Café itself served as a mechanism for mobilizing knowledge to the wider community. Building on the issue of credibility, relationships with trusted community providers can help enhance the perceived trustworthiness of VUC. Community partners have been shown to play an important role in improving the promotion of virtual health services for those facing systemic barriers to access [20,26]. While further work is required to build meaningful inroads with the larger community, this event served as a promising first step in raising awareness and co-designing some potential strategies for greater uptake.

Drawing on the lived experience of the World Café attendees, as well as their scope of practice, additional insights were gleaned related to those who face additional vulnerabilities, such as those who were unhoused or refugees. For instance, one VUC in the GTA created opportunities to use a strategically placed kiosk to increase accessibility to their service for those who did not have access to digital devices or a private place to receive care [27]. Similar recommendations for installing free, bookable and soundproof video booths at different community and hospital sites have been made elsewhere [28]. Future research exploring these options, which assesses the feasibility, acceptability and appropriateness of this strategy is warranted, but should also include measures of cost-effectiveness to sustain resources over the long-term at different sites and for different populations if found to be a viable solution.

While there were several identified gaps in care that persist with virtual models of care for EDPs, there were also opportunities that were discussed that could elevate standards of care. One noted issue was the quality of translation services available, which was not specific to the discussions regarding VUC *per se* (which employs a professional service to provide interpretation), but rather as a broader healthcare system concern. Although translation is available across many health settings, attendees discussed that the consistency of quality was lacking, particularly relating to the accuracy of the translated information. There is evidence that the use of professional interpreters results in high rates of patient satisfaction and offers the best route for communication between patients and providers [29]. However, there may be cultural nuances or medical terminology (even with native English speakers) that are difficult to convey, which could contribute to misunderstandings related to the patient’s history or treatment plan. To reduce the risks of these potential misunderstandings, one suggestion at the World Café that could lead to a greater understanding is to employ a teach-back method [30], which can be used to verify a patient’s understanding of their health information by having them repeat instructions received from their providers. This enables the provider to assess a patient’s understanding, correct misinterpretations, and modify the way the information is conveyed to ensure that it is clearly understood. The teach-back method has been widely used across different healthcare settings, including the ED, and generally has been found to be effective [30].

In terms of certain EDPs potentially being excluded from VUC, there is no data or anecdotal evidence that patients who are unhoused have used VUC provided at our home organization, which highlights the increased need for better outreach strategies. There is also a notable digital divide that exists with older adults, with data showing that only 12.5% of all VUC users at our organization are older adults (65 years and older). Given that older adults are heavy users of in-person EDs [31], representing between 20–40% of all visits depending on the location [32], there is an opportunity to explore ways to promote greater uptake within this population.

Opportunities discussed by World Café attendees included exploring the use of a self-triaging tool to help persons make better decisions on whether to use VUC or to opt for in-person care. For some models of virtual care, a virtual triage software is employed that relies on algorithms to assess the severity of a patient’s condition, and identify the presence of any ‘red flag’ or alarming symptoms or risk factors, which can then advise if a person should seek immediate care in the in-person ED [33]. There is evidence that the use of virtual triaging holds a number of benefits for clinicians, including streamlining patient-clinician communications, personalizing care delivery, aligning virtual care with clinical practice, and increasing impact and satisfaction derived from virtual care [33]. Similarly, an Ontario-based mixed-methods study assessing the virtual triaging system in a rural-setting as an alternative to going to the in-person ED found that patients valued the system’s accessibility, care quality and effectiveness [34]. Consequently, as awareness and uptake of VUC continues, there is likely a need to build in additional mechanisms that could capitalize on advances in artificial intelligence, such as the triaging interface, which may support health system navigation for patients. Regardless, it is essential to embed equity considerations into any such triaging system to ensure it helps reduce, rather than widen, the digital divide.

Identifying ways to offer strong integrated care to improve care transitions were noted to be of considerable importance for persons from EDPs. In some cases, this was about having a “warm handover” if the person is required to transition from a VUC visit to an in-person one. This includes facilitating their transition into the in-person ED and ensuring that their medical history from the VUC visit is accurately transferred and available to inform the current assessment. Although there are online portals that contain information that patients can access to better track their journey of care, these are not easily accessible due to some of the same challenges patients face in using online platforms (e.g., digital literary, non-English preference, cognitive impairments, etc.). Some system gaps highlighted were pertinent for patients experiencing substance misuse disorders or residents of long-term care homes. There may be an opportunity to create stronger ties with substance misuse detox centres and long-term care homes to minimize unnecessary transfers and in-person visits, and to increase access to clinicians for additional consultations for these patient populations. Overall, these described opportunities reinforce how integrated care systems can significantly contribute to the healthcare experience of EDPs by improving accessibility, availability, affordability, and acceptability [35,36]. Hence, there is a need for a clearer delineation of how VUC fits within the larger health and social care system in order to maximize its potential.

The findings from this work align with other recommendations on ways to achieve equitable access to telehealth, which includes adopting a patient-centred care design, creating culturally appropriate solutions, fostering trust between patients and providers, and ensuring confidentiality of patient information [37]. The use of the World Café approach was therefore highly suitable for advancing critical considerations for improving VUC access to EDPs since participatory design methods (co-design and user engagement) is a potential solution for planning culturally-competent virtual health services [38]. There is evidence that when end-users are properly engaged, this will lead to improvements in the user experience [39], as well as to telehealth care delivery [40], and contributes to capacity building [41] and health literacy [42]. Although not discussed, issues of sustainability of VUC (i.e., long-term funding) also requires continued reflection as well as its adaptability to meet the needs of various populations [43].

Despite having mechanisms in place to support participation in the World Café from participants with limited English proficiency (i.e., translation supports), we could not identify persons from this group to participate, even with targeted outreach with support by our community partners. Similarly, the majority of participants with lived experience were also persons serving in a professional capacity, and not all EDP groups were fully represented at the event (i.e., Indigenous, uninsured patients, refugees). However, our attendees represented a diverse range of roles and sectors (i.e., hospital, community, academic), and provided health and community support to many diverse communities (i.e., unhoused, Indigenous, 2SLGBTQIA + , disability, people living with mental health challenges or cognitive impairments [i.e., dementia]), which provided a number of important considerations relevant for addressing the various VUC needs of these EDPs.

## Conclusions

Virtual models of urgent care are becoming more standardized and serving a key component in a larger integrated healthcare system [44]. As with any advance in care, and particularly those that rely on technology as the primary mean of service delivery, there is a need to carefully reflect on how persons from diverse backgrounds may access and use these services to avoid widening existing health inequities. Given the potential of VUC to serve as a culturally safer and more accessible modality for EDPs [6,8], the World Café event served as a meaningful opportunity to exchange knowledge on health equity and to co-design community informed strategies for improved awareness and uptake of VUC by EDPs.
